# Genomic Balance: Two Genomes Establishing Synchrony to Modulate Cellular Fate and Function

**DOI:** 10.3390/cells8111306

**Published:** 2019-10-23

**Authors:** Justin C. St. John

**Affiliations:** The Mitochondrial Genetics Group, The Robinson Research Institute and The School of Medicine, Adelaide Health and Medical Sciences Building, The University of Adelaide, Adelaide, SA 5005, Australia; jus.stjohn@adelaide.edu.au

**Keywords:** Mitochondrial DNA, genomic balance, mtDNA copy number, tumour, stem cell, oocyte, assisted reproduction

## Abstract

It is becoming increasingly apparent that cells require cooperation between the nuclear and mitochondrial genomes to promote effective function. However, it was long thought that the mitochondrial genome was under the strict control of the nuclear genome and the mitochondrial genome had little influence on cell fate unless it was extensively mutated, as in the case of the mitochondrial DNA diseases. However, as our understanding of the roles that epigenetic regulators, including DNA methylation, and metabolism play in cell fate and function, the role of the mitochondrial genome appears to have a greater influence than previously thought. In this review, I draw on examples from tumorigenesis, stem cells, and oocyte pre- and post-fertilisation events to discuss how modulating one genome affects the other and that this results in a compromise to produce functional mature cells. I propose that, during development, both of the genomes interact with each other through intermediaries to establish genomic balance and that establishing genomic balance is a key facet in determining cell fate and viability.

## 1. Introduction

For many years, the nuclear and mitochondrial genomes were viewed and studied as independent entities. A transformation then took place that led to the concept of ‘nuclear-mitochondrial interaction’ primarily initiated by studies related to the role that the nuclear-encoded mitochondrial transcription and replication factors played in regulating mitochondrial transcription and replication [1,2]. In this case, the nuclear genome was not only regarded as being the major driving force, but, to a large extent, the only force at play. More recently, we have started to think about how the two genomes interact to potentially establish ‘Genomic Balance’. In this instance, the two genomes establish a degree of cooperation within the cell that they co-inhabit to promote effective function, and in a cell-specific manner. This cooperation results in the synchronisation of the two genomes to modulate energy metabolism and gene expression networks and pathways that are specific to each stage of development that the naïve and differentiating cell seeks to achieve. However, these interactions are complex and they appear to be highly dependent on the molecular regulatory mechanisms that also help to determine cellular fate. Consequently, genomic balance is likely to be under the influence of epigenetic control, which, in turn, can be mediated by the by-products of energy metabolism. This likely results in a tug of war between the genomic regulators operating between the two genomes that seek to mediate cellular fate, much in the same way as the ‘evolutionary intragenomic conflict’ was proposed in the 1990s [3].

## 2. The Nuclear Genome

The biparentally inherited human nuclear genome, in its haploid form, which was primarily observed in the mature oocyte, comprises approximately 3,234.83 Mega base pairs (Mb) of DNA; and 6,469.66 Mb in its diploid state, of which 1.5% contributes to the coding genes that give rise to proteins [4]. The remainder contributes to non-coding DNA regions that were once described as ‘junk’ DNA. However, an increasing proportion of non-coding DNA has important regulatory functions, such as small non-coding RNA genes, which include micro-RNAs, small nuclear RNAs, and small nucleolar RNAs; long non-coding RNA genes that include ribosomal RNAs; and, regulators of gene expression, such as epigenetic modifiers that define protein-encoding gene activity. Other regions act as regulators of DNA sequences, long-interspersed nuclear elements (LINES), short-interspersed nuclear elements (SINES), introns, and regions that will likely yield other functional roles that are currently under investigation.

## 3. The Mitochondrial Genome

**Figure 1:**
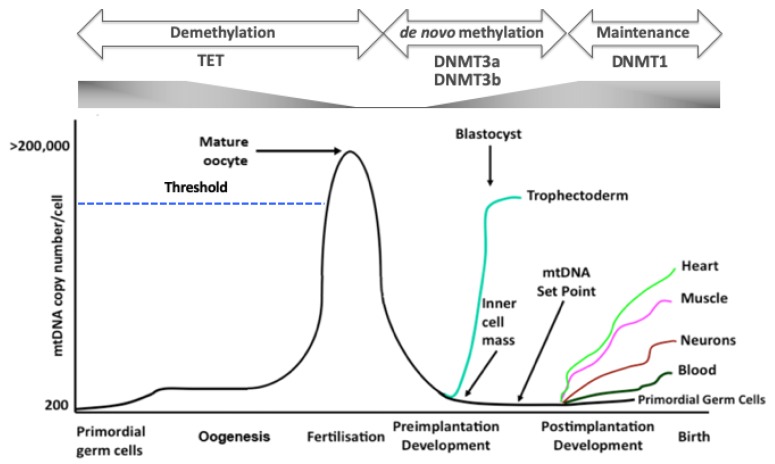
DNA methylation and mtDNA replication. Primordial germ cells, the very first germ cells laid down, possess very few copies of mtDNA. During oogenesis, mtDNA copy number increases exponentially to cross the threshold (broken blue line) that is required for the mature, metaphase II oocyte to complete fertilisation and preimplantation development. Throughout preimplantation development, mtDNA copy number decreases and mtDNA replication is then initiated at the blastocyst stage, but this is restricted to the trophectodermal cells. On the other hand, the cells of the inner cell mass continue to reduce mtDNA copy number. As a result, the developing embryo establishes the mtDNA set point, the founder molecules of mtDNA that contribute to the cells, tissues and organs of the foetus and offspring, which takes place before differentiation. Once naïve cells commit to a specific lineage, mtDNA replication takes place in a cell-specific manner, which allows cells to perform their specialized functions by allowing them to utilise oxidative phosphorylation (OXPHOS), if required. To this extent, cells (heart, muscle, neurons) that primarily have a high requirement for ATP through OXPHOS will have high mtDNA copy number and those with a low requirement for OXPHOS (blood) will tend to have lower mtDNA copy number and predominantly utilise glycolysis. Throughout development, there are synchronous changes to DNA methylation and gene expression. Translocation methylcytosine dioxygenases (TET) enzymes are responsible for erasing parental DNA methylation patterns through to the blastocyst stage. However, the de novo methyltransferases, for example DNMT3a and DNMT3b mediate de novo DNA methylation through preimplantation development and onwards. DNMT1 is then employed to maintain cell-specific DNA methylation profiles.

The mitochondrial genome, on the other hand, is a uniparentally inherited, double-stranded, circular, genome that is between 16.2 and 16.7 Kb in size across mammalian species [5]. It comprises 13 protein-encoding genes, 22tRNAs and two rRNAs, and it has two non-coding regions that are housed on the heavy and light strands. The non-coding regions comprise the D-loop, which contains two hypervariable regions and the control region where transcription and replication are initiated in the first instance on the heavy strand; and, one minor regulatory region that contains the light strand promoter region. Unlike the nuclear genome, cells have multiple copies of mitochondrial DNA (mtDNA) that range from as few as six copies in mature sperm that have fertilisation potential [6] to >200,000 copies in mature metaphase II (fertilisable, haploid) oocytes [7] (Figure 1). Indeed, the total mtDNA content of metaphase II oocytes is approximately 3,313.8Mb, which suggests that mtDNA is an equal or greater than equal DNA contributor at the time of fertilisation.

## 4. Cooperation between the Two Genomes

The nuclear and mitochondrial genomes both cooperate by encoding subunits of the electron transfer chain, a key energy generating apparatus that is located in the inner mitochondrial membrane [8]. The electron transfer chain generates the vast majority of ATP required by cells with complex functions through the biochemical process of oxidative phosphorylation (OXPHOS) [8]. Indeed, the electron transfer chain is the only cellular apparatus that is encoded by the two genomes, and, whilst the nuclear genome is the major contributor in terms of encoded gene products, mutation or deletion to one or more of the mtDNA-encoded genes can lead to severe cellular, tissue, or organ dysfunction that can be lethal [9]. Furthermore, organisation and assembly of the electron transfer chain requires nuclear-encoded factors to chaperone subunits that are encoded by both genomes into their rightful positions [10,11], demonstrating the complexity of the cooperation.

Nevertheless, intergenomic communication, the study of the interactions between the two genomes has extensively focussed on how the nuclear-encoded mtDNA transcription and replication factors translocate to the mitochondrion and interact to drive first transcription and then replication of the mitochondrial genome [1,2]. Other studies have focussed on the dual role played by the mitochondrial specific transcription factors, and specifically TFAM, in initiating transcription [12,13]; and, acting as a packaging protein [14,15], given its two high mobility group-box domains that endows it with relatively close homology to the histone family of proteins [16]. Indeed, there are a host of factors that are necessary for driving these processes; and mutation to some of these genes, such as the mtDNA-specific polymerase, polymerase gamma (*POLG*) [17,18] and *TFAM* [19,20], has led to severe phenotypes, where, as with mutation to the mitochondrial genome, cells, tissues and organs are affected in a similar manner [9]. However, many of these factors are unique to mitochondrial transcription and replication, but arise from distant ancestral systems that are indicative of the mitochondrion’s bacterial origins [21]. For example, the processivity of POLG, is only specific to the replication of mtDNA [22]. Indeed, in terms of mtDNA replication, the nucleus accommodates the mitochondrial genome by encoding factors specific to polymerase (POLG and POLG2) [22], helicase (TWINKLE) [23], topoisomerase (TOP1MT) [24], and single stranded binding (MTSSB1) [25] activities, as well as the initiation of mtDNA replication (TFAM) [12,13]. This previously contributed to the view that the nuclear genome regulates the mitochondrial genome and there is little or no influence from the mitochondrial genome on the nuclear genome.

## 5. Synchrony of the Two Genomes During Development

Both genomes are strictly regulated during the early stages of development. The nuclear genome undergoes frequent division as cells of the newly formed embryo cleave, which is aided by cells primarily utilising glycolysis for energy production by the blastocyst stage [26]. Consequently, replication of the nuclear genome is supported by a faster supply of lower levels of energy to promote this activity during early development. At the same time, the mtDNA copy number is reduced in each newly formed cell [27,28] as a result of there being no replication of mtDNA until post-gastrulation [29] (Figure 1); and due to the active secretion of the mitochondrial genome into its neighbouring environment [30]. These changes are mirrored by changes in the patterns of de novo DNA methylation that take place during development [31,32], as depicted in Figure 1. Indeed, a key event takes place at or around gastrulation when mtDNA copy number has been further reduced to establish the mtDNA set point. The mtDNA set point is characterised by mtDNA copy number being at its lowest levels, and, in naïve cells, gives rise to the founder populations of mtDNA molecules. These copies are then replicated and, thus, contribute to the foetus’s cells, tissues, and organs, and ultimately those of the offspring [33,34]. Shortly after, there is a change from de novo DNA methylation to maintenance DNA methylation [31,32]. During oogenesis, the opposite takes place, whereby global DNA demethylation is mirrored by exponential increases in mtDNA copy number [35,36] (Figure 1). This ensures that the primordial germ cells mature into fertilisable, metaphase II oocytes, and they possess sufficient copies of mtDNA to support developmental events post-fertilisation [7,28,37,38,39,40], i.e., this is considered to be a genomic investment in mtDNA copy number to support subsequent developmental events [41]. Indeed, oocytes with too few copies of mtDNA, to a greater extent, either fail to fertilise or arrest during preimplantation development [7,28].

The numbers of mtDNA copy in a cell are frequently indicative of a cell’s stage of development or the fate of a cell. For example, a naïve, pluripotent cell, such as an embryonic stem cell or a fully dedifferentiated induced pluripotent stem cell, will have low mtDNA copy number [42,43], and, at the same time, *POLG* will be extensively DNA methylated, primarily within a CpG island in its second exon [44,45]. Indeed, it is possible to determine each cell type’s capacity for mtDNA replication by expressing mtDNA copy number for a defined cell type as a ratio of its methylated state within *POLG*. To this extent, assaying 5-methylcytosine (5mC; active DNA methylation) and 5-hydroxymethylcytosine (5hmC; transient methylation/demethylation) through, for example, an antibody pulldown approach and determining their respective levels of interaction within the CpG island through real time PCR, enables the levels of mtDNA copy number in relation to the availability of *POLG* for transcription and ultimately protein expression to be determined [45]. As a result, cells that are pluripotent or multipotent in nature group together [45]. In a similar fashion, tumour cells and differentiated cells cluster into distinct groups. Interestingly, induced pluripotent cells, which have not completed the process of dedifferentiation, exhibit different patterns of mtDNA copy number and DNA methylation within *POLG* and, thus, mtDNA replicative capacity. They are unable to complete the process of differentiation when induced to do so and they fail to effectively replicate their mtDNA copy number [43]. This suggests that their nuclear and mitochondrial genomes are not acting in synchrony. However, when these cells are treated with a DNA demethylation agent, such as 5-Azacytidine, they faithfully replicate their mitochondrial genomes, as they undergo differentiation and meet the key mtDNA replication checkpoints [43] that are in place at key stages of differentiation [29].

The outcomes above suggest that the balance between the two genomes can be reset, so that their interactions are restored to promote cell function. Therefore, if we accept that the concept of genomic balance is in play, what are the consequences of upsetting or modulating the balance in favour of either the nuclear or the mitochondrial genome? We will now investigate this, firstly, in cells that give rise to disease and, then, in the context of cells that are presumed to be of normal function.

## 6. Modulating the Nuclear Methylome

**Figure 2:**
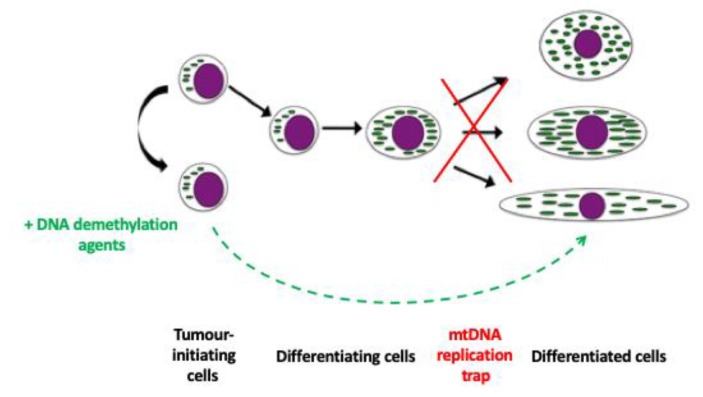
The mtDNA replication trap. Tumour-initiating cells are frequently unable to complete differentiation. Consequently, they are trapped between a naïve and a differentiated state. However, some DNA demethylation agents act by resetting the epigenome and mtDNA copy number in these cells, which then allows for the cell to differentiate into its mature phenotype with both the nuclear and mitochondrial genomes acting in synchrony.

Tumour cells that exhibit a stem cell-like nature, such as Glioblastoma multiforme (GBM) cells [46,47], can give rise to severe forms of cancer with a low rate of survival [48]. Unlike stem cells, they are unable to complete the process of differentiation and replicate their mtDNA in an asynchronous manner [47] and, consequently, they are trapped at an early stage of differentiation [49] (Figure 2). However, when GBM cells are treated with 5-Azacytidine or another DNA demethylation agent, vitamin C, they initiate and progress differentiation [47] (Figure 2). Indeed, they revert from a hypermethylated to a more hypomethylated state, as demonstrated by global DNA methylation analysis [50]. In line with this outcome, a number of the mtDNA replication factors also undergo DNA demethylation with the most striking effects for *POLG* and *TOP1MT* [50] and, consequently, they replicate mtDNA [47,50]. Interestingly, global DNA demethylation also mediates DNA demethylation of the mitochondrial genome [50].

In recent times, there has been renewed interest in whether the mitochondrial genome is DNA methylated. Previously, it was assumed that the mitochondrial genome was unmethylated [51]. However, although conflicting data now exist regarding the presence of mtDNA methylation [52,53,54,55], it appears that the mitochondrial genome is subject to methylation at CG, CHG, and CHH sites [56]. Furthermore, it remains to be determined whether DNA methylation influences transcriptional activity of the mitochondrial genome or whether it plays other, to be identified, roles. This is especially the case, as the mechanisms influencing mtDNA methylation requires further elucidation, given that there is uneven DNA methylation across the mitochondrial genome [50]. In the context of the concept of genomic balance, mtDNA methylation adds a degree of complexity to the extent that the mediators of DNA methylation may act on each genome separately or in tandem, a factor that remains to be determined, given that both the DNMT enzymes and DNA demethylation enzymes, namely the ten-eleven translocation methylcytosine dioxygenases (TET), have been identified in mitochondria [52,57]. Nevertheless, DNA methylation is likely to be involved in establishing and regulating genomic balance between the two genomic compartments of the cell. In this respect, the demethylation of the mitochondrial genome is likely to be linked with mtDNA replication rather than transcription, as DNA demethylation does not appear to lead to the anticipated increases in transcription of the mtDNA protein-encoding genes, as shown in tumour-initiating cells [50]. However, the changes in mtDNA methylation were in synchrony with the demethylation that was observed for the nuclear-encoded mtDNA replication factors and a very large cohort of nuclear-encoded developmental genes [50]. This reflects a commitment by both genomes to establish synchrony that would, in turn, promote the replication of the mitochondrial genome. In this case, demethylation of the nuclear-encoded mtDNA replication factors results in their increased expression, whilst the mitochondrial genome is demethylated to ensure that more template is available for replication.

## 7. Modulating mtDNA Copy Number 

As previously discussed, cells acquire specific numbers of mtDNA copy to provide the appropriate levels of OXPHOS-derived ATP to perform their specialised functions. Cells can be depleted of their mtDNA to varying degrees through the use of mtDNA depletion agents in order to determine the effects of altering mtDNA copy number in cells. Typically, these have included ethidium bromide [58] and 2′-3′-dideoxycytidine (ddC) [59], and they are introduced to cells whilst in culture. The steady-state reduction of mtDNA copy number on GBM cells through ddC induces dedifferentiation to more of a pluripotent state [47]. Cells depleted to 50% and 70% of their original mtDNA content have the propensity to undergo neural differentiation and increase mtDNA copy number in a synchronous manner [47]. However, cells that were depleted to greater degrees are unable to mediate these changes. At the same time, cells depleted to 50% of their mtDNA content exerted greater tumorigenic potential when introduced into immune deficient mice. These tumours grew at faster and more aggressive rates than non-depleted cells. However, cells depleted to very low levels formed significantly fewer tumours and, when tumours formed, they took significantly longer. When compared with tumours that were derived from non-depleted cells, tumours derived from the more depleted cell cohorts exhibited very different global DNA methylation and nuclear gene expression profiles [60]. The affected genes were associated with gene networks and pathways related to behaviour, nervous system development, cell differentiation, and regulation of transcription and cellular processes. Interestingly, in all tumours, mtDNA copy number was restored to the pre-depletion levels [47]; and was in line with a significant modulation of a large cohort of the mtDNA replication factors and correlated with changes in DNA methylation of *POLG* [50].

Similar outcomes have been observed when multiple myeloma cells were introduced into immune deficient mice [61]. Tumours formed from cells possessing their full complement of mtDNA and those depleted to 10% of their original content. However, cells that were depleted to almost no mtDNA content resulted in no tumours forming. Nevertheless, tumours that were derived from cells depleted to 10% of their original content grew at a slower rate and their localisation was less focal than for non-depleted cells. In each case, mtDNA copy number was restored in tumours that formed [61]. Again, this highlights the level of cooperation between the two genomes to ensure optimal cell function is established to support tumorigenesis and it is reinforced by outcomes from tumour cells that do not have the propensity to restore mtDNA copy number, namely rho-0 cells. In a model of osteosarcoma, rho-0 cells were able to acquire mtDNA from their neighbouring micro-environment, most likely the stroma, to promote tumorigenesis [61]. In human cells that acquire mouse mtDNA, this divergent form of mtDNA is unlikely to have replicative capacity when driven by the human mtDNA replication machinery [62], and, thus, incomplete tumours form [61]. Nevertheless, the presence of low levels of mtDNA is sufficient for promoting tumorigenesis. However, mouse tumour cells that acquire neighbouring mouse mtDNA form fully developed and efficient tumours due to the greater compatibility between the two genomes enabling more efficient mtDNA replication and, thus, repopulation of the cell with more compatible populations of mtDNA [63].

## 8. Altering the mtDNA Genotype

In the tumour model of osteosarcoma, it has been shown using three different tumour-initiating cell lines, in which each line possesses the same nuclear genotype but a different mitochondrial genotype, that they induce marginal differences in time to tumorigenesis [61]. However, each of the three lines possessed different levels of mtDNA copy number at early and late stages of tumorigenesis. Furthermore, there were differences in global nuclear gene expression patterns. Interestingly, of the key genes that are associated with the initiation of osteosarcoma, there was a core group of genes that was commonly differentially expressed between early and late stages of tumorigenesis. However, there were also mtDNA genotype differences again highlighting the impact that the mitochondrial genome can have on tumour gene expression profiles and formation.

Other studies using tumour cells, which have replaced their mitochondrial genomes with those harbouring mtDNA mutations that are associated with mtDNA disease, have shown that the genetic rearrangement induces changes to the epigenetic status of the cell by modulating histone modulators [64]. However, it remains to be conclusively shown whether the mitochondrial genome harbouring the mutation or the mutation itself induced the epigenetic changes. In a series of mouse embryonic stem cell models consisting of several cell lines that possessed the same nuclear genotype but different mtDNA genotypes, there were significant effects on pluripotent nuclear gene expression and DNA methylation profiles in undifferentiated cells [65]; and also global DNA methylation profiles [66]. These effects also reflected on the gene expression profiles of these cells during differentiation [66]. This demonstrates that mtDNA can influence cells from very early stages, which results in profound effects later during differentiation and likely during development. Indeed, it was evident that the mtDNA genotypes differentially regulated a key metabolite from the TCA cycle, namely α-ketoglutarate, which acts as a co-factor with the TET proteins to mediate the transition from 5mC to 5hmC [66]. Consequently, a two-way compensatory approach seems to operate between the two genomes within a cell to ensure that the synchrony of the two genomes is balanced to promote cell differentiation and that the resultant cells are viable. These outcomes are further reflected in conplastic mouse studies where the same nucleus interacts with different mtDNA genotypes. In this instance, the mtDNA background affected a number of metabolic parameters, including insulin signalling and obesity, and ageing [67].

## 9. Potential Implications for Assisted Reproduction

One of the most contentious areas associated with influencing genomic balance is likely to be assisted reproduction, as the outcomes could have profound effects on offspring health, well-being, and survival. Whilst natural conception and in vitro fertilisation result in considerable changes to the genetic make-up of the individual, mtDNA replication [13,68] and de novo DNA methylation profiles [69,70] during very early development appear to follow prescribed pathways that, in turn, result in developmental milestones being met, which determine the degree of an offspring’s developmental progression. However, the more invasive assisted reproductive technologies, for example cytoplasmic transfer and nuclear transfer, can result in the aberrant transmission of mtDNA to the offspring, frequently, with offspring inheriting two populations of mtDNA, namely from the cytoplasmic [71,72] or nuclear donor [73,74], respectively; and, aberrant patterns of de novo and re- methylation early during development [75]. With somatic cell nuclear transfer (SCNT), a number of very diverse developmental disorders have been reported [76] that likely have their origins associated with epigenetic defects [70,77]. However, a number of these offspring exhibit phenotypes that are similar to those that were reported in mtDNA disease [76]. In terms, of cytoplasmic transfer, some developmental disorders have been reported [71], but the overall sample size and number of reports are too limited to determine the cause of the phenotypic abnormalities.

In the context of nuclear transfer, a somatic cell undergoes reprogramming of its nucleus in order that it adopts a more pluripotent state with the ultimate aim of matching that of the fertilised oocyte [78]. This is primarily mediated by factors that are present in the cytoplasm of the enucleated recipient oocyte. However, this can also be enhanced by using epigenetic modifying agents, for example 5-Azacytidine (DNA methyl-transferase inhibitor) and Trichostatin A (histone deacetylase inhibitor; TSA), prior to or post-nuclear transfer in a large number of species, including cattle [79,80], pigs [81,82], rabbits [83], and sheep [84]. In many respects, this represents the most extreme form of re-establishing genomic balance. The failure to completely reprogram the somatic cell results in the aberrant regulation of mtDNA replication during preimplantation development with the continued expression of the key mtDNA replication factors, namely POLG and TFAM [85]. The somatic cell’s mtDNA, which is carried over into the recipient oocyte, can be preferentially selected and account for 0 to 59% of the offspring’s total mtDNA content [86]. The mechanisms for selection in this process are unknown, but the close proximity of the donor cell’s mtDNA to the nucleus during the first round of mtDNA replication could account for this. The interesting question in this context is whether this is an attempt to re-establish genomic balance or whether the two genomes are simply out of synchrony with each other. Nevertheless, this highlights the importance of the long process that the oocyte undergoes, as it progresses from the primordial germ cell stage through to the metaphase II stage in readiness for fertilisation or to host a somatic cell.

In relation to oocytes that were deficient in mtDNA, i.e., oocytes that do not surpass the putative threshold for efficient fertilisation outcome [7,37,38], their supplementation with genetically identical mtDNA from sister oocytes induces interesting outcomes in terms of readdressing genomic balance [87]. Firstly, they result in increased developmental rates to the blastocyst stage. Secondly, they have significantly increased mtDNA copy number due to a mtDNA replication event that takes place between fertilisation and the two-cell stage [87] that arises from changes to the DNA methylation of specific CpG sites within a large CpG island of *POLG,* which, in turn, alters the expression of this gene [88]. Finally, there were shifts in gene expression from the mature, metaphase II oocyte as a result of mtDNA supplementation through each stage of preimplantation development up to and including the blastocyst stage [87,88,89]. In a model of bovine SCNT, the depletion of donor cell mtDNA prior to SCNT led to differences in nuclear gene expression by the blastocyst stage of development when compared with blastocysts that were derived from non-mtDNA-depleted donor cells and were further enhanced by the use of TSA [90]. However, the addition of oocyte-derived mtDNA into this model (minus TSA) resulted in different gene expression profiles with increased expression levels for genes associated with OXPHOS, cell cycle, and DNA repair [91]. Nevertheless, a comparison of the gene networks and pathways in blastocysts that were generated by SCNT in the presence of TSA and those derived from additional mtDNA alone indicated the benefits of mtDNA-supplementation alone through increased glycolytic activity and reduced embryonic cell death. This suggests that the addition of mtDNA was able to promote reprogramming at the expense of a reprogramming agent.

## 10. Defining Genomic Balance 

**Figure 3:**
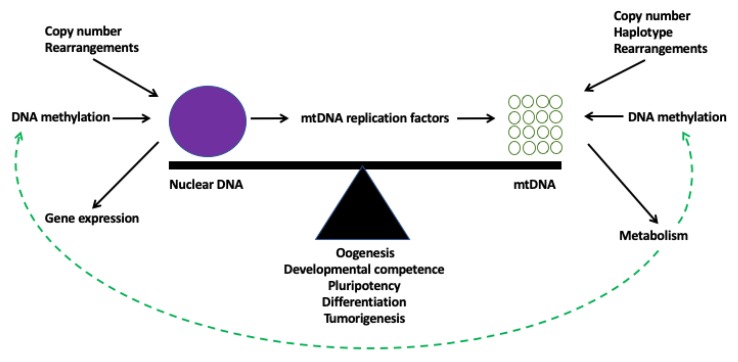
Model of genomic balance. At any given stage in development, for a cell to be ultimately functional, it needs to establish genomic balance. There is a continual flow of regulatory information between the nucleus and the mitochondrial genome that ensures there are sufficient copies of mtDNA to meet a cell’s functional requirements for ATP through OXPHOS. At the level of the nuclear genome, genomic balance is mediated by epigenetic changes, for example the levels of DNA methylation, which control gene expression. Other factors include DNA rearrangements, such as mutations and deletions, and copy number variants. At the level of the mitochondrial genome, the choice of cellular metabolism will affect mtDNA copy number, which, in turn, is aided by the cell’s mtDNA genotype. As a result, metabolic factors are released which can modulate DNA methylation and other epigenetic modifiers that regulate both the nuclear and mitochondrial genomes.

It would appear that both genomic compartments within a cell establish a relationship that enables them to effectively cooperate for the benefit of that cell’s specific function(s). Figure 3 highlights some of the key interactions and the influencing factors. The interactions appear to be highly sophisticated and will likely be a compromise resulting from the cell’s state of metabolism at any given time responding to the cell’s desired fate. Likewise, the metabolic factors that are generated by the cell will mediate the epigenetic profile of the cell, through, for example, co-factors that regulate the levels of DNA methylation. Again, the metabolic fate of the cell will be at the behest of the amount of mtDNA copy present in the cell, which, in turn, is likely governed by the cell-specific levels of DNA methylation regulating the expression of the mtDNA-specific replication factors, of which *POLG* and *TOP1MT* appear to be two key candidates. These changes will fluctuate at different stages of development and during differentiation, but will set the pathway that will result in the fate of the terminally differentiated cell. These are not only key developmental events for the somatic cell but also for gametes, which are the ultimate determiners of the individual’s phenotype once they have, as sperm and oocytes, combined following fertilisation. Indeed, both of the gametes go through significant waves of de/methylation through their differentiation and perturbing these effects through, for example, oocyte reconstruction could account for the anomalies that are associated with some assisted reproductive technologies. Perhaps, if we were to view tumour formation in this manner, we might regard it as a pathway of choice that is available to the naïve cell but, in a multitude of circumstances, is avoided until the pathway is triggered.

## 11. Conclusions

In this review, I have sought to discuss the existence of a relationship between the two genomic compartments of the cell that is likely to be an essential determiner of cell fate and stabiliser of the phenotype of the mature, functional cell. This relationship draws on the regulators of gene expression and mtDNA replication to provide synchrony that ensures a balance between the states of the nuclear and mitochondrial genomes in order that they behave in a specific manner at any given stage during development and differentiation to determine fate and phenotype. This relationship will be dynamic during early stages of development, as naïve cells respond to environmental cues and stimuli. However, the relationship will be in a steadier-state once the cell has achieved its endpoint fate whether as a functional somatic cell that might mediate, for example, neural activity; or, a tumour cell that would give rise to a severe, if not fatal, outcome.
